# Fungi in perennial ice from Scărișoara Ice Cave (Romania)

**DOI:** 10.1038/s41598-018-28401-1

**Published:** 2018-07-04

**Authors:** Traian Brad, Corina Itcus, Madalina-Denisa Pascu, Aurel Perșoiu, Alexandra Hillebrand-Voiculescu, Lavinia Iancu, Cristina Purcarea

**Affiliations:** 1 Institute of Biology, 296 Splaiul Independenţei, 060031 Bucharest, Romania; 2 Emil Racoviţă Institute of Speleology, Clinicilor 5, Cluj-Napoca, 400006 Romania; 3 National Institute of Research and Development for Biological Sciences, 296 Splaiul Independenţei, 060031 Bucharest, Romania; 4 Ștefan cel Mare University, Suceava, Universității 13, 720229 Suceava, Romania; 5 Emil Racoviţă Institute of Speleology, 31 Frumoasă St, 010986 Bucharest, Romania

## Abstract

Screening of 1,000-years old ice layers from the perennial ice block of Scărișoara Ice Cave (NW Romania) revealed the presence of fungal communities. Using culture-dependent methods and molecular techniques based on DGGE fingerprinting of 18S rRNA gene fragments and sequencing, we identified 50 cultured and 14 uncultured fungi in presently-forming, 400 and 900 years old ice layers, corresponding to 28 distinct operational taxonomic units (OTUs). The dominant ice-contained fungal OTUs were related to Ascomycota, Basidiomycota and Cryptomycota phyla. Representatives of Mucoromycota and Chytridiomycota were also isolated from recent and 400 years old ice samples. The cryophilic *Mrakia stokesii* was the most abundant fungal species found in the cave ice samples of all prospected ages, alongside other cryophilic fungi also identified in various glacial environments. Ice deposits formed during the Little Ice Age (dated between AD 1,250 and 1,850) appeared to have a higher fungal diversity than the ice layer formed during the Medieval Warm Period (prior to AD 1,250). A more complex fungal community adapted to low temperatures was obtained from all analyzed ice layers when cultivated at 4 °C as compared to 15 °C, suggesting the dominance of cold-adapted fungi in this glacial habitat. The fungal distribution in the analyzed cave ice layers revealed the presence of unique OTUs in different aged-formed ice deposits, as a first hint for putative further identification of fungal biomarkers for climate variations in this icy habitat. This is the first report on fungi from a rock-hosted cave ice block.

## Introduction

Over the last decades, investigation of icy environments became a major scientific direction to unravel the presence, adaptation mechanisms and role of ice-contained microorganisms^1–3^. In spite of low temperatures, life is not absent in frozen environments, and organisms, primarily bacteria, have found various ways to survive and colonize different icy habitats^4–8^. Such habitats include polar and high mountain ice caps, alpine glaciers, permafrost and rock-hosted cave glaciers. The later – hereafter called ice caves – refer to ice accumulations in caves, formed as seepage water freezes to form underground perennial ice bodies, some of them thousands of years old^9^. Apart from surface glaciers (polar or alpine) that form as snow slowly metamorphoses into ice, most of cave ice deposits form from water that has percolated through soil and rock and ponded for days through months before freezing. As such, they could host most peculiar microbial assemblages, and are among the poorest investigated cold habitats^10^. The presence of bacteria in an alpine ice cave was first mentioned by Margesin and collaborators^11^ who isolated and characterized a psychrophilic *Arthrobacter psychrophenolicus* strain from the cave non-icy sediments. Bacterial strains belonging to the genera *Pseudomonas, Acidovorax* and *Brevundimonas* were isolated from ice deposits of Oregon Cascades lava tube^12^, while several profiles of prokaryotic communities from Antarctic volcanic ice caves (i.e, caves carved at the interface between rock and glacier surface) were recently described^13^. Moreover, the occurrence of Archaea in the sediments of an Austrian ice cave was also mentioned^14^. In Scărișoara Ice Cave (NW Romania), our previous investigations revealed the presence of diverse bacteria in recent ice stalagmites^15^, including phototrophic microbes in sunlight-exposed ice, and various types of cultured^16^ and uncultured^17^ bacteria in ice layers up to 900 years old from the perennial ice block harbored in the cave.

In addition to prokaryotes, icy environments also contain complex fungal communities^3,18,19^. In the Arctic, various yeast species were detected in sub-glacial water, sea ice and sea water^20^, permafrost^21^, glacier surface and ice cores from Northern Greenland, at depths of about 2 km^22–24^. Fungi were also discovered in various Antarctic habitats such as permafrost^25,26^, decaying wood^27^, lake^28^, sub-glacial^29^ and sea water^30^, and ice cores as deep as 3.5 km, corresponding to 1 to 2 million years old ice^31^. While information on psychrophilic fungi from South American, Asian and Himalayan icy environments is rather scarce, several reports mentioned their presence in glacial habitats from Europe, including sediments from alpine glaciers and permafrost^18,32,33^, sub-glacial sediments and water, as well as glaciers ice cores^28,34^.

Ice cave habitats, mainly screened for prokaryotic organisms, also revealed the presence of uncultured and cultured fungi in ice sediments. Fungal strains were isolated from an Arctic glacier cave located in Svalbard archipelago^21^, and the diversity of uncultured fungi was reported from an Antarctic volcanic ice cave^35^. Meanwhile, very few reports refer to fungi from perennial ice deposits in alpine caves. Among these, two psychrophilic *Cryptococcus* spp. strains and one heterobasidiomycete were isolated from an Austrian ice cave and characterized^36^. In Scărișoara Ice Cave, the presence of fungi in different layers of the perennial ice block was revealed by 18S rRNA gene amplification^17^.

While the presence of fungi in caves was reported worldwide^37^, no studies of the possible use of fungi as biomarker of past climatic changes have been carried out so far. Building on the unique records of past climatic and environmental changes derived from the ice in Scărişoara Ice Cave (Romania), identification of fungal diversity throughout the cave ice block could lead to a novel microbial climatic proxy. In this context, the current data, representing the first report on fungal diversity in an alpine ice cave, led to the identification of cultured and uncultured fungi in perennial ice layers up to 900 years old from Scărişoara Ice Cave, based on DGGE analysis and sequencing of 18S rRNA gene amplicons.

## Results and Discussion

### Cave ice fungal community based on DGGE analysis

**Figure 1:**
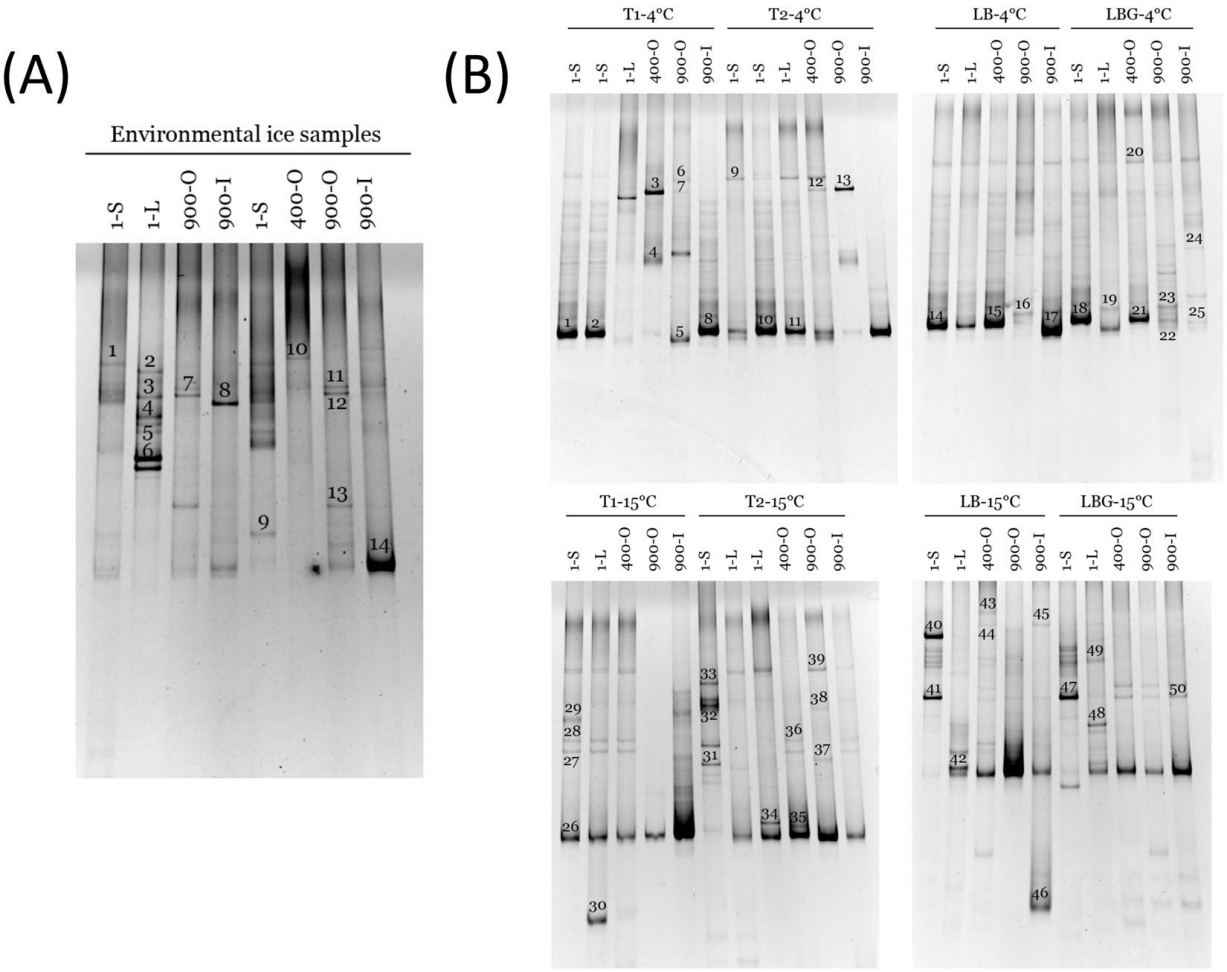
DGGE profile of 18S rRNA gene amplicons from Scărișoara ice samples. Fungal 18S rRNA gene fragments were amplified from 1-S, 1-L, 400-O, 900-O and 900-I (**A**) environmental ice samples, and (**B**) the corresponding enrichment cultures obtained in T1, T2, LB and LBG media at 4 °C and 15 °C, and analyzed by DGGE as previously described^16^. The number on each sample amplicon corresponds to the fungal sequence (Table 1).

Denaturing gradient gel electrophoresis (DGGE) profiles of 18S rRNA gene fragments retrieved from the 1-S, 1-L, 400-O, 900-O and 900-I cave ice samples (Fig. 1A) and from their enrichment cultures (Fig. 1B) revealed the presence of complex fungal communities throughout the cave ice block, with a relatively different profile of fungal amplicons from ice samples collected from different layers and corresponding cultured microbiota.

Amplicon based analysis of the DGGE gels (data not shown) revealed a relatively higher number of fungal OTUs in the recent ice deposits 1-S and 1-L (13.22 ± 3.41) as compared to older ice strata (9.33 ± 3.94). Also, the fungal community profiles of the 400-O ice sample and enrichments appeared to be more complex (average OTUs number 10.44 ± 3.61) than those of the 900-O and 900-I samples (average OTUs number 7.11 ± 2.85). These latter ice deposits were formed during the Medieval Warm Period (MWP; AD 800–1,250), that corresponded to warmer and wetter climate conditions than those formed 400 years ago (sample 400-O) during the cold and dry Little Ice Age (LIA), that lasted between AD 1,250 and 1,850^9,38,39^. In addition to temperature, the higher fungal diversity identified in the 400-O ice sample could be related to the higher organic content of this ice layer as compared to that of the clear ice sample 900-I^16^. However, the cultured fungal communities at both 4 °C and 15 °C from 400-O ice sample appeared to be more diverse than those from the 900 years old samples (900-O and 900-I), independent of their organic content. Moreover, no significant differences in fungal amplicon profiles were observed between the 900 years old ice layers of high (900-O) and low (900-I) organic content. In this case, the cultured fungal community at 4 °C contained a larger number of amplicons (12.70 ± 3.76) relative to that grown at 15 °C (8.70 ± 3.73). As expected, the cold-active fungal community from Scărișoara Ice Cave enrichments appeared to be more diverse than that growing at higher temperature, similar to the cultured bacterial community from the same habitat^16^.

### Fungal diversity in cave ice

**Figure 2:**
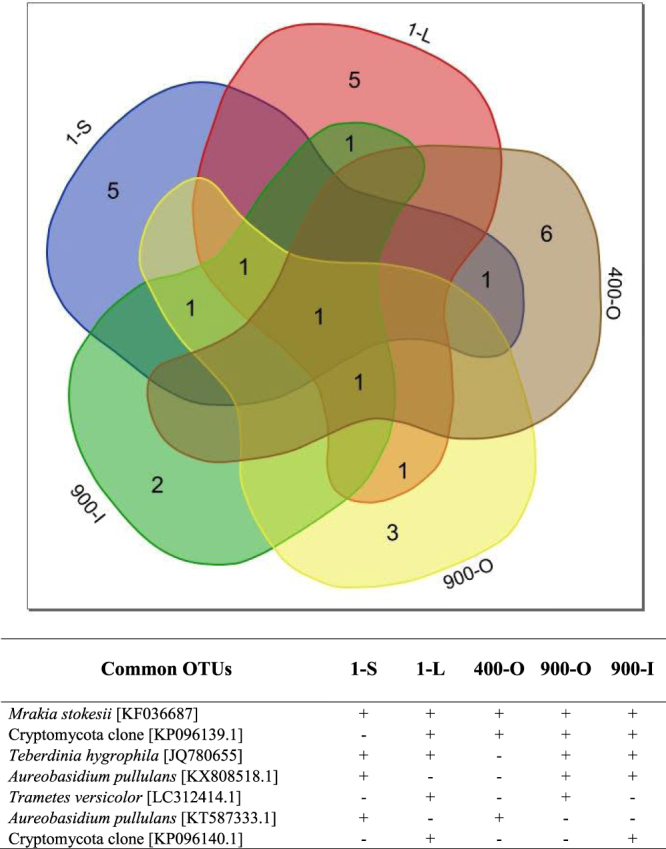
Distribution of fungal OTUs in cave ice chronosequence. VENN diagram indicates the number of distinct and shared fungal OTUs in Scărişoara ice samples 1-S, 1-L, 400-O, 900-O and 900-I. The GenBank closest match and distribution (presence/absence: +/−) of 7 fungal strains common to different cave ice layers are indicated in the table.

Sequencing of the 64 DGGE-extracted 18S rRNA amplicons, among which 14 from ice samples and 50 from enrichment cultures, led to the identification of 28 different fungal OTUs in the perennial ice from Scărişoara Ice Cave (Table 1). Ten OTUs corresponded to uncultured fungi from cave ice samples, while 23 OTUs resulted from enrichment cultures at 4 °C (16 OTUs) or 15 °C (13 OTUs). The distribution of the identified fungal OTUs in different layers of the cave ice block highlighted 21 specific OTUs for individual ice samples, while 7 fungal OTUs are shared between different ice layers (Fig. 2). Several fungal OTUs from the cave ice block appeared to be homologous to other cold environments-originated strains (Table 1). Among these, the psychrophilic yeast^40^ *Mrakia stokesii* was the most represented species (14 OTUs) in the analyzed environmental ice samples and in both 4 and 15 °C enrichment cultures, considering that *Mrakia gelida* is a synonymous species of *M. stokesii*^41^. Another cryophilic yeast, *Teberdinia hygrophila*, identified in alpine fen soil from Caucasus Mountains^42^, was present in the clear ice sample 1-L, and recovered from enrichment cultures of both presently-forming and 900 years old ice performed at 4 °C (1-S) and 15 °C (1-S, 900-O and 900-I) (Fig. 2).

**Table 1:** Closest match of fungal OTUs from Scărişoara ice cave.

DGGE gel band		Sample code	Sequence accession no.	GenBank closest match	Affiliation (phylum)	Identity %
Cave ice	1	SG.1-Sa	KY614706	Uncultured fungus clone B5 18S rRNA gene [JN054661.1]	Unidentified	91
	2	SG.1-La	KY614707	Uncultured fungus isolate DGGE gel band 7 18S rRNA gene [FJ914608.1]	Unidentified	91
	3	SG.1-Lb	KY614708	Uncultured Cryptomycota clone CES109_15 18S rRNA gene [KP096139.1]	Cryptomycota	98
	4	SG.1-Lc	KY614709	*Teberdinia hygrophila* isolate 229 small subunit rRNA gene [JQ780655.1]	Ascomycota	99
	5	SG.1-Ld	KY614710	*Mrakia gelida* strain CBS5272 18S rRNA gene [KF036685.1]	Basidiomycota	98
	6	SG.1-Le	KY614711	Uncultured Cryptomycota clone CES507_8 18S rRNA gene [KP096140.1]	Cryptomycota	96
	7	SG.900-Oa	KY614712	*Mrakia stokesii* strain CBS5917 18S rRNA gene [KF036687.1]	Basidiomycota	90
	8	SG.900-Ia	KY614713	*Mrakia stokesii* strain CBS5917 18S rRNA gene [KF036687.1]	Basidiomycota	98
	9	SG.1-Sb	KY614714	*Valsaria lopadostomoides* strain VIQ 18S rRNA gene [KP687972.1]	Ascomycota	83
	10	SG.400-O	KY614715	Uncultured Cryptomycota clone CES109_15 18S rRNA gene [KP096139.1]	Cryptomycota	98
	11	SG.900-Ob	KY614716	*Mrakia stokesii* strain CBS5917 18S rRNA gene [KF036687.1]	Basidiomycota	99
	12	SG.900-Oc	KY614717	*Brachyconidiella monilispora* 18S rRNA gene [AY342015.1]	Ascomycota	79
	13	SG.900-Od	KY614718	*Aureobasidium pullulans* strain TSTRWY1 18S rRNA gene [KX808518.1]	Ascomycota	99
	14	SG.900-Ib	KY614719	*Aureobasidium pullulans* strain TSTRWY1 18S rRNA gene [KX808518.1]	Ascomycota	93
Cultured fungi	1	SM.1-S.T1-4a	KY614552	*Mrakia stokesii* strain CBS5917 18S rRNA gene [KF036687.1]	Basidiomycota	99
	2	SM.1-S.T1-4b	KY614553	Uncultured Mucorales clone BFC113 18S rRNA gene [GU305985.1]	Mucoromycota	97
	3	SM.400-O.T1-4a	KY614554	Uncultured fungus clone CV1_B2_34 18S rRNA gene [AY821997.1]	Unidentified	94
	4	SM.400-O.T1-4b	KY614555	*Glaciozyma antarctica* strain cHCR18v 18S rRNA gene [KY306721.1]	Basidiomycota	97
	5	SM.900-O.T1-4a	KY614556	*Aureobasidium pullulans* strain TSTRWY1 18S rRNA gene [KX808518.1]	Ascomycota	93
	6	SM.900-O.T1-4b	KY614557	Uncultured Cryptomycota clone CES507_8 18S rRNA gene [AY934720.1]	Cryptomycota	96
	7	SM.900-O.T1-4c	KY614558	*Mrakia stokesii* strain CBS5917 18S rRNA gene [KF036687.1]	Basidiomycota	99
	8	SM.900-I.T1-4	KY614559	*Aureobasidium pullulans* strain TSTRWY1 18S rRNA gene [KX808518.1]	Ascomycota	99
	9	SM.1-S.T2-4a	KY614560	*Aureobasidium pullulans* strain TSTRWY1 18S rRNA gene [KX808518.1]	Ascomycota	95
	10	SM.1-S.T2-4b	KY614561	*Teberdinia hygrophila* isolate 229 small subunit rRNA gene [JQ780655.1]	Ascomycota	97
	11	SM.1-L.T2-4	KY614562	*Mrakia stokesii* strain CBS5917 18S rRNA gene [KF036687.1]	Basidiomycota	99
	12	SM.400-O.T2-4	KY614563	Uncultured Cryptomycota gene for 18S rRNA [AB971034.1]	Cryptomycota	93
	13	SM.900-O.T2-4	KY614564	Uncultured Cryptomycota clone CES109_15 18S rRNA gene [KP096139.1]	Cryptomycota	97
	14	SM.1-S.LB-4	KY614565	*Aureobasidium pullulans* strain TSTRWY1 18S rRNA gene [KY781746.1]	Ascomycota	99
	15	SM.400-O.LB-4	KY614566	Uncultured Cryptomycota clone CES109_15 18S rRNA gene [KP096139.1]	Cryptomycota	98
	16	SM.900-O.LB-4	KY614567	Uncultured Cryptomycota clone CES109_15 18S rRNA gene [KP096139.1]	Cryptomycota	99
	17	SM.900-I.LB-4	KY614568	Uncultured Chytridiomycota clone T2P1AeA04 18S rRNA gene [GQ995409.1]	Chytridiomycota	96
	18	SM.1-S.LBG-4	KY614569	*Mrakia stokesii* strain CBS5917 18S rRNA gene [KF036687.1]	Basidiomycota	99
	19	SM.1-L.LBG-4	KY614570	*Trametes versicolor* gene for 18S rRNA, strain: K-41 [LC312414.1]	Basidiomycota	97
	20	SM.400-O.LBG-4a	KY614571	*Aureobasidium pullulans* culture-collection 18S rRNA gene [KT587333.1]	Ascomycota	99
	21	SM.400-O.LBG-4b	KY614572	*Acidomyces acidothermus* strain MH1112 18S rRNA gene [JQ172747.2]	Ascomycota	90
	22	SM.900-O.LBG-4a	KY614573	*Aureobasidium pullulans* strain TSTRWY1 18S rRNA gene [KX808518.1]	Ascomycota	98
	23	SM.900-O.LBG-4b	KY614574	*Periconia macrospinosa* isolate WC10G1 18S rRNA gene [KU981158.1]	Ascomycota	91
	24	SM.900-I.LBG-4a	KY614575	Uncultured Cryptomycota clone CES304_1 18S rRNA gene [KP096143.1]	Cryptomycota	96
	25	SM.900-I.LBG-4b	KY614576	Uncultured Cryptomycota clone CES109_15 18S rRNA gene [KP096139.1]	Cryptomycota	89
	26	SM.1-S.T1-15a	KY614577	Uncultured Mucor isolate DGGE gel band K19a 18S rRNA gene [JX560304.1]	Mucoromycota	97
	27	SM.1-S.T1-15b	KY614578	*Aureobasidium pullulans* strain TSTRWY1 18S rRNA gene [KX808518.1]	Ascomycota	99
	28	SM.1-S.T1-15c	KY614579	*Mrakia stokesii* strain CBS5917 18S rRNA gene [KF036687.1]	Basidiomycota	98
	29	SM.1-S.T1-15d	KY614580	*Teberdinia hygrophila* isolate 229 18S rRNA gene [JQ780655.1]	Ascomycota	98
	30	SM.1-L.T1-15	KY614581	*Thelebolus sp*. 19 BI 15-3-1 18S rRNA gene [GU004225.1]	Ascomycota	98
	31	SM.1-S.T2-15a	KY614582	*Aureobasidium pullulans* culture-collection 18S rRNA gene [KT587333.1]	Ascomycota	93
	32	SM.1-S.T2-15b	KY614583	Uncultured Mucor isolate DGGE gel band K19a 18S rRNA gene [JX560304.1]	Mucoromycota	99
	33	SM.1-S.T2-15c	KY614584	*Aureobasidium pullulans* strain TSTRWY1 18S rRNA gene [KX808518.1]	Ascomycota	98
	34	SM.1-L.T2-15	KY614585	Uncultured eukaryote clone 18S_S7_clon30 18S rRNA gene [HQ873429.1]	Unidentified	95
	35	SM.400-O.T2-15a	KY614586	*Thelebolus microsporus* partial 18S rRNA gene, strain R-43237 [FR717359.1]	Ascomycota	98
	36	SM.400-O.T2-15c	KY614588	Uncultured fungus isolate DGGE gel band F5 18S rRNA gene [KX274111.1]	Unidentified	96
	37	SM.900-O.T2-15a	KY614589	*Teberdinia hygrophila* isolate 229 small subunit rRNA gene [JQ780655.1]	Ascomycota	98
	38	SM.900-O.T2-15b	KY614590	*Trametes versicolor* gene for 18S rRNA, strain: K-41 [LC312414.1]	Basidiomycota	97
	39	SM.900-O.T2-15c	KY614591	*Mrakia stokesii* strain CBS5917 18S rRNA gene [KF036687.1]	Basidiomycota	99
	40	SM.1-S.LB-15a	KY614592	*Mrakia stokesii* strain CBS5917 18S rRNA gene [KF036687.1]	Basidiomycota	99
	41	SM.1-S.LB-15b	KY614593	*Mrakia stokesii* strain CBS5917 18S rRNA gene [KF036687.1]	Basidiomycota	99
	42	SM.1-L.LB-15	KY614594	*Mrakia stokesii* strain CBS5917 18S rRNA gene [KF036687.1]	Basidiomycota	98
	43	SM.400-O.LB-15a	KY614595	*Mrakia stokesii* strain CBS5917 18S rRNA gene [KF036687.1]	Basidiomycota	96
	44	SM.400-O.LB-15b	KY614596	Uncultured Cryptomycota clone CES109_15 18S rRNA gene [KP096139.1]	Cryptomycota	97
	45	SM.900-I.LB-15a	KY614597	*Teberdinia hygrophila* isolate 229 18S rRNA gene [JQ780655.1]	Ascomycota	99
	46	SM.900-I.LB-15b	KY614598	*Aureobasidium pullulans* strain TSTRWY1 18S rRNA gene [KX808518.1]	Ascomycota	100
	47	SM.1-S.LBG-15	KY614599	*Teberdinia hygrophila* isolate 229 18S rRNA gene [JQ780655.1]	Ascomycota	99
	48	SM.1-L.LBG-15a	KY614600	Uncultured Cryptomycota clone CES507_8 18S rRNA gene [KP096140.1]	Cryptomycota	94
	49	SM.1-L.LBG-15b	KY614601	Uncultured eukaryote gene for 18S rRNA [AB902208.1]	Unidentified	94
	50	SM.900-I.LBG-15	KY614602	Uncultured Cryptomycota clone CES507_8 18S rRNA gene [KP096140.1]	Cryptomycota	97

Sequence code indicates the ice sample (1-S, 1-L, 400-O, 900-O, 900-I), growth medium (T1, T2, LB, LBG), growth temperatures (4 °C or 15 °C), and DGGE band number (Fig. 1).

The ice-originating fungal strain homologous to the Cryptomycota clone KP096139.1 was present in all age ice layers (1-L, 400-O, 900-O, and 900-I samples), while *Teberdinia hygrophila, Aureobasidium pullulans, Trametes versicolor* and another clone belonging to phylum Cryptomycota (accession no. KP096140.1) were found in recent ice and 900 years old ice layers (Fig. 2). The *Aureobasidium pullulans* (accession no. KT587333.1) strain was identified in both the 1-S and 400-O samples originating from high organic content ice deposits (Fig. 2). *Thelebolus* species, also identified in our study (SM.1-L.T1-15), were described from Antarctic lakes^43^. A close relative of the SM.400-O.LBG-4b enrichment culture from 400 years old ice was *Acidomyces acidothermus*, previously identified in acidic soils from Czech Republic and Iceland^44^. Sequences of more ubiquitous fungi (*Brachyconidiella monilispora*, *Valsaria lopadostomoides*, *Periconia macrospinosa* and *Trametes versicolor*) were also identified in presently-forming and 900 years old ice samples, and recovered in all enrichment cultures at both 4 °C and 15 °C. Thirteen OTUs corresponded to unidentified fungi (Table 1), suggesting a higher diversity of these eukaryotic microorganisms in Scărişoara ice block.

**Figure 3:**
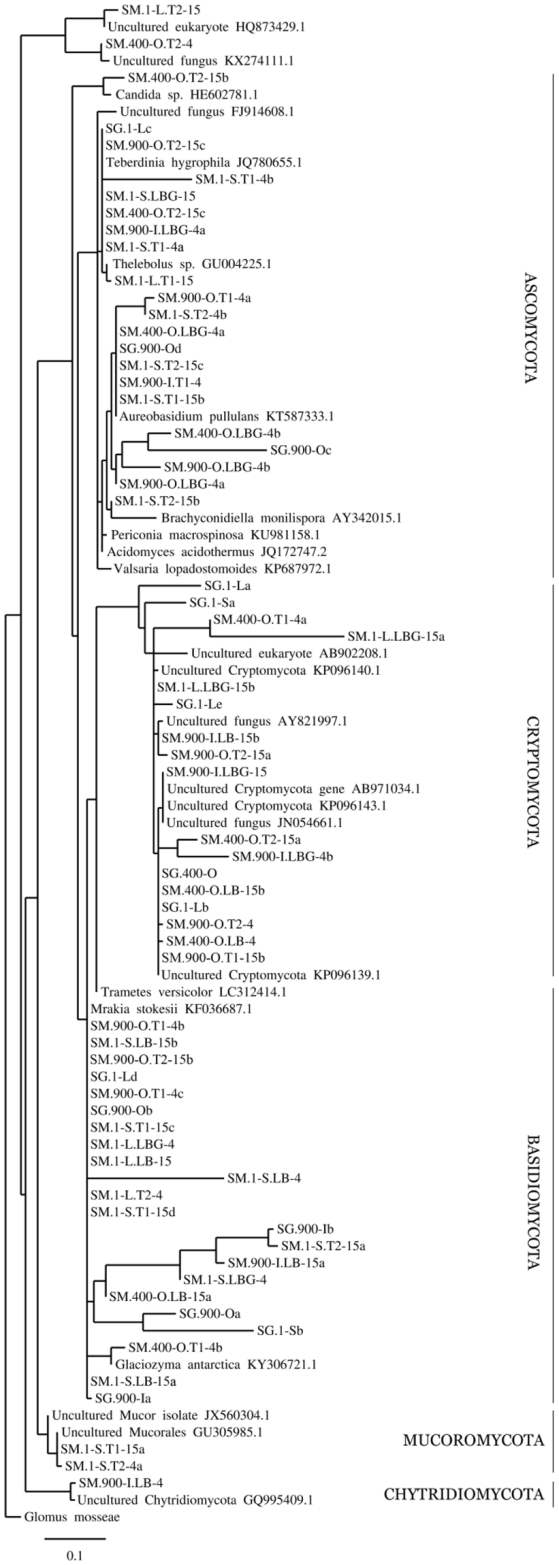
Phylogenetic tree of fungi from ice samples collected from Scărişoara Ice Cave and from the corresponding enrichment cultures, based on 18S rDNA. The cave ice 18S rRNA gene sequences and closest fungal match (Table 1) were used for the phylogenetic tree construction, using *Glomus mosseae* [NG017178]^45^ as outgroup for tree rooting.

The fungal strains were homologous to known OTUs belonging to five phyla (Table 1). A phylogenetic analysis, using *Glomus mosseae* [NG017178]^45^ as outgroup, revealed clearly separated corresponding clades of the fungal tree (Fig. 3). Among these, representatives of the phylum Ascomycota prevailed (25 sequences), followed by those belonging to Basidiomycota (17 sequences), Cryptomycota (13 sequences), Mucoromycota (3 sequences) and Chytridiomycota (1 sequence) phyla. Previous investigations of fungi from glacial environments indicated the dominance of basidiomycetous yeasts. These fungi were identified in Antarctic ice^1^, Arctic ice^21,46^, and mountain glaciers in the Apennines^32^. Fewer studies described the occurrence of ascomycetous fungi in frozen environments, including Arctic ice from Svalbard Archipelago^20^ and volcanic ice caves on Mt. Erebus in Antarctica^35^. Our data showed that the ice cave environment selected for particular types of cryophilic fungi. Members of the phylum Cryptomycota were identified in recent ice (1-L) and 400-O samples and recovered in enrichment cultures of ice of all ages. Species belonging to Cryptomycota phylum were identified also in Arctic and Antarctic ice cores from Svalbard^47^ and from Lake Vostok at 3.5-km depth^31^, respectively. Viable fungal spores were isolated from Antarctic ice cores more than 1.5 million years old and from Greenland ice cores 140,000 years old^48,49^. Likewise, the identified fungi in the perennial ice from Scărişoara cave could originate from the surface and trapped within the ice layers that succeeded every year, in the presence of organic debris accumulated on the bottom of supraglacial pond formed during summer time on top of the ice block, while engulfed permanently by the ice during winter^50,51^.

In Scărişoara Ice Cave, new layers of ice form on at the surface every year, trapping the microbial communities within the ice, with no further contact with the outer surface of the ice block^50^. In these settings, the microbial communities may form spores that allow them to endure for centuries^22,26^. Alternatively, these microorganisms may survive deep in the ice in micro-spaces surrounding various types of debris insoluble in frozen water. A thin film of unfrozen water may form around mineral particles allowing microorganisms to perform metabolic reactions in icy habitats^4^. To decipher their metabolic status, in the case of culturable fungi found in the cave ice block, further investigations are needed using high-throughput RNA sequencing, a study that is presently underway. Diversity of cultured and uncultured fungi present in perennial ice collected from layers of different ages in Scărişoara Ice Cave can provide evidence for climate change effects. Melting of the cave perennial ice due to global warming could release viable microorganisms trapped for the last hundreds of years in the ice block^51^. These microbes could be of significant value both for basic science and applications in bionanotechnologies and cryopreservation^52^.

Ice melting could lead to revival of microorganisms considered extinct, and isolation and characterized of entirely new taxa entrapped in ice. In our study, 19 OTUs shared less than 97% identity with the closest known relatives in GenBank database, constituting putative novel strains. Such new fungi may have major influences of the existing environment by interfering with strains of same species that have evolved and adapted to different environmental conditions, while the new arrivals have rested for centuries of even millennia. The emergence of the new microbes could have a major impact on other organisms, including humans, possibly generating disease outbreaks. Among these, *Aureobasidium pullulans*, also detected in Scărişoara ice samples, is known to produce various skin infections^53^.

Our data not only provided a first glimpse on the fungal diversity in different aged ice layers of an ice cave, a pioneering study in this type of habitat, but led to isolation of a series of cultured cold-active fungal strains of putative applicative potential. The presence of some dissimilar fungal OTUs in different aged ice layers of Scărişoara ice block could constitute promising leads for future identification of fungal climate indicators. A deeper sequencing of an extended ice block chronosequence using NGS, currently underway, should provide more insight into the ice cave fungal diversity and the possible use of these microorganisms as a proxy for climate variation.

## Material and Methods

### Ice sampling

Scărişoara Ice Cave harbors one of the largest (100,000 m^3^) and the oldest (>10,000 years old) perennial cave ice blocks in the world^9^. The cave is located in Bihor Mountains, NW Romania, (46°29′23″N, 22°48′35″E), at an altitude of 1,165 m^51^. The ice block is formed of ice layers deposited annually by freezing of percolating water, containing debris with microorganisms carried to the cave from the surface^54–56^. As a result of this depositional mechanism, each annual stratum is formed by an upper layer of clear ice and a bottom layer of debris-rich ice. Further, two mechanisms are responsible for the formation of extremely rich layers of debris: during periods of enhanced melting, several annual layers of ice are melted and the debris they contain is concentrated in one single, thicker layer, while inflow of water from the surface during wetter periods lead to the formation of similarly thick layers of debris. The two can be distinguished by their content, with the latter being richer in macrofossils washed-in from the surface^37^. Five ice samples of variable age and sediment content were collected aseptically from newly formed ice (1-S and 1-L) corresponding to direct (S) and indirect (L) sunlight exposed areas, 400 (400-O) and 900 (900-O and 900-I) years old ice deposits, from organic-rich (O) and clear ice (I) layers of the ice block^16^.

### PCR-DGGE

The enrichment cultures of melted ice samples in T1, T2, LB and LBG media^57^, and the DNA extractions from both the environmental ice samples and enrichments were performed as previously described^16^. The 18S rRNA gene fragments were amplified using the fungal-specific primers FF390 (CGA TAA CGA ACG AGA CCT) and FR1-GC (the GC clamp is underlined, CCC CCG CCG CGC GCG GCG GGC GGG GCG GGG GCA CGG GCC G AIC CAT TCA ATC GGT AIT)^58^. PCR amplification was performed in a 50-μl reaction mixture containing 110 ng cave-ice DNA, 100 pmol of both forward and reverse primers, 0.2 mM dNTP, and 1.25 U MyTaq HS DNA Polymerase (Bioline, London, UK), in the presence of 1× MyTaq Reaction Buffer. Amplification was performed in a Thermal Cycler C1000™ (Bio-Rad Laboratories, Hercules, CA, USA), using an initial denaturation of 95 °C for 8 min, followed by 30 cycles of 95 °C for 30 s, 50 °C for 45 s and 72 °C for 2 min, and a final elongation step at 72 °C for 10 min.

DGGE was carried out using a DGGE-4801-220 system (C.B.S. Scientific Company Inc., Del Mar, CA, USA). PCR products were loaded onto 8% (wt/vol) polyacrylamide (ratio of acrylamide to bisacrylamide 37.5:1) gels. A 20 to 35% linear denaturing gradient was used, where 100% denaturant was defined as 7 M urea and 40% (vol/vol) formamide. Electrophoresis was performed in 1 × TAE buffer (40 mM Tris–acetate, 1 mM Na-EDTA; pH 8.0) at 200 V and 60 °C for 4 h. The gels were stained in 1 × TAE buffer containing 1 μg ml^−1^ ethidium bromide, and the amplicon profile of the DGGE gels was used to compare the fungal diversity in different environmental samples and enrichment cultures, using statistical tests.

### Sequencing and data analysis

18S rDNA amplicons were excised from the DGGE gels, incubated 48 h at 4 °C in 20 µl sterile water, and re-amplified by PCR as described above. Sanger sequencing was performed (Macrogen, Amsterdam, Netherlands) using the FF390 primer^58^. The DNA sequences were edited using CodonCode Aligner and BioEdit for eliminating the sequencing errors. The closest match of each OTU was determined using the BLAST-NCBI Megablast algorithm and the nucleotide collection database^59^. Sequence alignment was performed using ClustalW and the phylogenetic tree was generated using the maximum likelihood analysis, using the www.phylogeny.fr platform^60^. The 18S rDNA sequences were deposited in the GenBank DNA database under accession numbers KY614706-KY614719 (uncultured fungi) and KY614552-KY614602 (cultured fungi).
